# Enumerating Virus-Like Particles and Bacterial Populations in the Sinuses of Chronic Rhinosinusitis Patients Using Flow Cytometry

**DOI:** 10.1371/journal.pone.0155003

**Published:** 2016-05-12

**Authors:** Jessica A. P. Carlson-Jones, James S. Paterson, Kelly Newton, Renee J. Smith, Lisa M. Dann, Peter Speck, James G. Mitchell, Peter-John Wormald

**Affiliations:** 1 School of Biological Sciences, Flinders University, Adelaide, South Australia, Australia; 2 Department of Surgery-Otolaryngology Head & Neck Surgery, The University of Adelaide, South Australia, Australia; Institut National de la Santé et de la Recherche Médicale, FRANCE

## Abstract

There is increasing evidence to suggest that the sinus microbiome plays a role in the pathogenesis of chronic rhinosinusitis (CRS). However, the concentration of these microorganisms within the sinuses is still unknown. We show that flow cytometry can be used to enumerate bacteria and virus-like particles (VLPs) in sinus flush samples of CRS patients. This was achieved through trialling 5 sample preparation techniques for flow cytometry. We found high concentrations of bacteria and VLPs in these samples. Untreated samples produced the highest average bacterial and VLP counts with 3.3 ± 0.74 x 10^7^ bacteria ml^-1^ and 2.4 ± 1.23 x 10^9^ VLP ml^-1^ of sinus flush (n = 9). These counts were significantly higher than most of the treated samples (p < 0.05). Results showed 10^3^ and 10^4^ times inter-patient variation for bacteria and VLP concentrations. This wide variation suggests that diagnosis and treatment need to be personalised and that utilising flow cytometry is useful and efficient for this. This study is the first to enumerate bacterial and VLP populations in the maxillary sinus of CRS patients. The relevance of enumeration is that with increasing antimicrobial resistance, antibiotics are becoming less effective at treating bacterial infections of the sinuses, so alternative therapies are needed. Phage therapy has been proposed as one such alternative, but for dosing, the abundance of bacteria is required. Knowledge of whether phages are normally present in the sinuses will assist in gauging the safety of applying phage therapy to sinuses. Our finding, that large numbers of VLP are frequently present in sinuses, indicates that phage therapy may represent a minimally disruptive intervention towards the nasal microbiome. We propose that flow cytometry can be used as a tool to assess microbial biomass dynamics in sinuses and other anatomical locations where infection can cause disease.

## Introduction

Chronic rhinosinusitis (CRS) is a common disease amongst the human population, and there is increasing evidence to show that microorganisms are involved in the inflammation of the sinus mucosal layer leading to exacerbation of the disease [1–3]. It is well established that the healthy sinus is not sterile, but is colonised by a diverse community of microorganisms [1, 2, 4, 5]. These microorganisms exist in the sinuses within exopolysaccharide biofilms, the presence of which leads to difficulties in treating the disease [6–12]. As antibiotics are a common treatment option for CRS patients, there is concern surrounding the growing antimicrobial resistance [13, 14]. Bacteria within these biofilms are able to secrete a polysaccharide matrix that acts as a protective barrier against host defences and antimicrobial agents [15]. This protective barrier makes it difficult when it comes to treating CRS with antibiotics. An alternative treatment is phage therapy which utilises specific bacteriophages (phage) that infect and kill pathogenic bacteria [16]. Trials of bacteriophage cocktails consisting of multiple types of phage, on sheep models of sinusitis have proven to be effective in eliminating *S*. *aureus* in biofilms and its free floating form [17]. Through the use of these phage cocktails, the development of phage resistant bacteria is reduced [18]. Knowledge of population abundance of bacteria and phage in the sinuses is important for the development of appropriate phage concentrations for use in this therapy [19].

Flow cytometry has been used as a method for enumerating heterotrophic bacteria and virus-like particles (VLPs) in environmental samples for decades [20–22]. This non-culture based technology is a quick, inexpensive way to rapidly enumerate a large number of cells and particles to provide data without the enrichment bias culturing introduces [20]. This technique yields highly reproducible counts and enumeration of VLPs at a concentration that would be too low for transmission electron microscopy (TEM) [20]. Here, we investigate methods to enumerate bacteria and VLPs within sinus flush fluid samples and present the first data, produced using flow cytometry, describing bacterial and VLP abundance within the maxillary sinuses of CRS patients. We, therefore, aim to measure the variation in abundance of bacteria and VLPs in the sinuses of CRS patients to determine if they are present at the same level among patients.

## Materials and Methods

### Ethics Statement

Maxillary sinus flush fluid samples were obtained from nine patients diagnosed with CRS in accordance to criteria defined by the Chronic Rhinosinusitis Task Force [23]. This study was approved by The Queen Elizabeth Hospital Human Research Ethics Committee, reference number: HREC/13/TQEHLMH/49. All nine patients involved in the study gave written consent prior to their sinus surgery. All sinus flush fluid samples were collected by the senior author (P.J.W) during the patient’s endoscopic sinus surgery. Due to the highly invasive nature of the operating procedure, healthy controls were not ethically justifiable, nor are they relevant to the question of how much abundance variation is there among infected patients.

#### Sample collection

Immediately after opening the maxillary sinus, approximately 5 ml of sterile saline was used to flush the sinus and re-collected in sterile specimen containers. Volume of flush fluid collected ranged from approximately 2 to 4 ml. Once samples were collected, they were transported on ice ready for immediate fixation with glutaraldehyde (0.5% final concentration) on ice in the dark, then snap freezing in liquid nitrogen and storage at -80°C until analysis [21].

### Sample Preparation

Five sample preparation techniques for flow cytometry were investigated. Fixed sinus flush fluid samples were thawed before each treatment was applied.

#### Sputasol

Sputasol was made using 0.02 μm filtered MilliQ water according to the manufacturer’s instructions (Oxoid). Equal volumes of Sputasol and fixed sinus sample were mixed together then incubated at 37°C for 15 minutes.

#### Methanol

Methanol, 0.2 μm filtered, was added to fixed sinus samples to a final concentration of 20% [24]. Samples were incubated at 37°C for 15 minutes then sonicated for 30 seconds in a SoniClean^™^ sonicating bath (Model 160TD, 60 W, 50/60Hz).

#### Potassium citrate

Potassium citrate tribasic solution (1M, Sigma) was added to the fixed sample to a 1% final concentration [24]. The sample was then vortexed for 30 seconds [24].

#### Sodium pyrophosphate

Sodium pyrophosphate solution was added to 100 μl of fixed sample to a final concentration of 10 mM [24]. Samples were vortexed for 1 minute and incubated at room temperature for 15 minutes [24]. Samples were then sonicated for 30 seconds in a SoniClean^™^ sonicating bath (Model 160TD, 60 W, 50/60Hz).

#### Untreated

Fixed samples were diluted in 0.2μm filtered TE buffer (10 mM Tris, 1 mM EDTA, pH 7.4) without pre-treatment [21].

### Flow Cytometric Analysis

Bacterial and VLP populations present in sinus flush fluid were identified and enumerated using a BD ACCURI C6 flow cytometer (Becton Dickinson). Samples using each extraction technique were run in triplicate for each patient. Samples were diluted (1:100) in 0.2 μm filtered TE buffer, stained with the DNA-binding dye SYBR-I Green (1:20,000 final dilution; Molecular Probes) then incubated at 80°C in the dark for 10 minutes [21]. For each preparation method control samples were generated prior to each flow cytometry session. These samples were prepared in the same manner as patient samples in 0.9% sterile saline, the same concentration used to flush patient sinuses. These samples were used to eliminate background artefacts introduced during sample preparation.

Samples were analysed using an Accuri C6 flow cytometer (Accuri C6) and BD ACCURI CFlow software. All samples were run for 2 minutes at machine fluidics setting of fast, with the threshold set to FL-1 (green fluorescence). As a control, 1 μm diameter fluorescent beads (Molecular Probes) were used. Beads were added to each sample to a final concentration of 10^5^ beads ml^-1^ [25]. The beads allow for flow cytometric parameters to be normalised according to bead fluorescence and concentration, and to give an indication of viral and bacterial cell size [25]. Phosphate-buffered saline (PBS) was used as sheath fluid for flow cytometry analysis. For each sample, green fluorescence, side angle light scatter and forward angle light scatter were recorded.

### Data Analysis

Flow cytometry data was analysed using FlowJo software (Tree Star, Inc.). VLP and bacterial populations were categorised based on variations in side scatter, a representation of cell size, and SYBR Green fluorescence, an indication of nucleic acid content [20, 21, 26]. For some patients, only one bacterial population was observed, whereas others showed multiple. Therefore, one overall bacterial population was created to remain consistent across all patient samples.

Rank abundance plots were generated for bacterial and VLP concentrations using all method triplicates and their averages to distinguish between any patient groupings formed on abundance. Comparisons between bacterial and VLP abundances for each treatment method were made using the statistical analysis program SPSS version 22 (IBM Corp. Released 2013. IBM SPSS Statistics for Windows, Version 22.0. Armonk, NY: IBM Corp.) using the Wilcoxon signed-rank test. Statistical significance between treatments was considered when p < 0.05.

## Results

### Flow Cytometric Analysis

**Fig 1:**
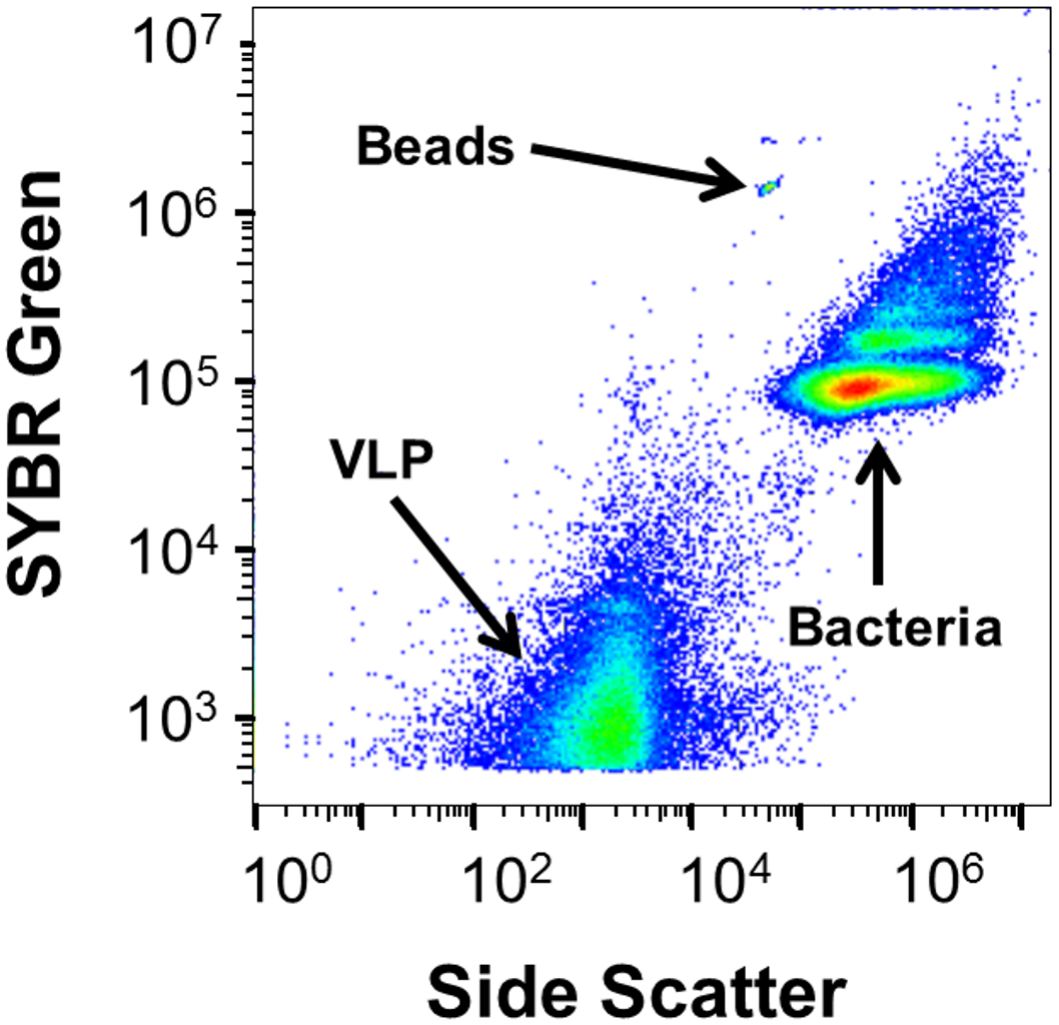
Bacterial and VLP identification using side-scatter and green fluorescence. Representative cytogram shows the VLP and bacterial populations in a patient’s untreated sinus wash.

Cytograms showed discrete bacterial and VLP populations present within the sinus fluid of CRS patients (Fig 1). Some patients exhibited one distinct bacterial population, whereas others patients exhibited up to four (Fig 1). Variations in bacterial and VLP abundance were observed between patients regardless of the treatment method used on the samples. Mean bacterial and VLP abundances for each treatment method are shown in Table 1.

**Table 1:** Mean concentration of bacteria and VLP per ml of sinus flush fluid for each treatment method tested. Error represents standard error of the mean.

Treatment	Bacteria ml^-1^ (±SE)	VLP ml^-1^ (±SE)
Untreated	3.3 x 10^7^ (7.4 x 10^6^)	2.4 x 10^9^ (1.2 x 10^9^)
Sodium pyrophosphate	2.9 x 10^7^ (6.7 x 10^6^)	2.0 x 10^9^ (9.9 x 10^8^)
Sputasol	2.2 x 10^7^ (6.5 x 10^6^)	1.8 x 10^9^ (9.1 x 10^8^)
Methanol	2.0 x 10^7^ (4.3 x 10^6^)	2.2 x 10^9^ (9.9 x 10^8^)
Potassium citrate	1.9 x 10^7^ (4.6 x 10^6^)	2.2 x 10^9^ (1.1 x 10^9^)

### Bacterial Sample Preparation Method Optimisation

For patient samples, the mean bacterial abundance for untreated samples was the highest of all treatments, with 3.3 ± 0.74 x 10^7^ cells ml^-1^ (n = 27; Table 1; S1 Table). The lowest mean abundance was for potassium citrate treated samples with 1.9 ± 0.46 x 10^7^ cells ml^-1^ (n = 27; Table 1; S1 Table). In testing the differences between treatments, the untreated and sodium pyrophosphate samples yielded significantly higher bacterial abundance than potassium citrate (p < 0.001 and p < 0.001), methanol (p = 0.002 and p < 0.001) and Sputasol (p = 0.003 and p < 0.001). There was no significant difference in bacterial abundance between sodium pyrophosphate treated and untreated samples (p = 0.39).

### VLP Sample Preparation Method Optimisation

VLP mean abundance for the untreated method was the highest for all patient samples with 2.4 ± 1.2 x 10^9^ cells ml^-1^ (n = 27; Table 1; S2 Table). Sputasol treated patient samples yielded the lowest VLP abundances with 1.8 ± 0.91 x 10^9^ cells ml^-1^ (n = 27; Table 1; S2 Table). Untreated samples yielded significantly higher VLP abundances than potassium citrate (p = 0.031), methanol (p = 0.010), and Sputasol (p = 0.019) treated samples. There was no significant difference between VLP abundance for untreated samples and sodium pyrophosphate treated samples (p = 0.08). Sodium pyrophosphate also did not yield significantly higher VLP abundances than potassium citrate (p = 0.44) and methanol (p = 0.53) treated samples. There was, however, a significant difference between VLP abundances for sodium pyrophosphate and Sputasol (p = 0.008).

### Bacterial Rank Abundance

**Fig 2:**
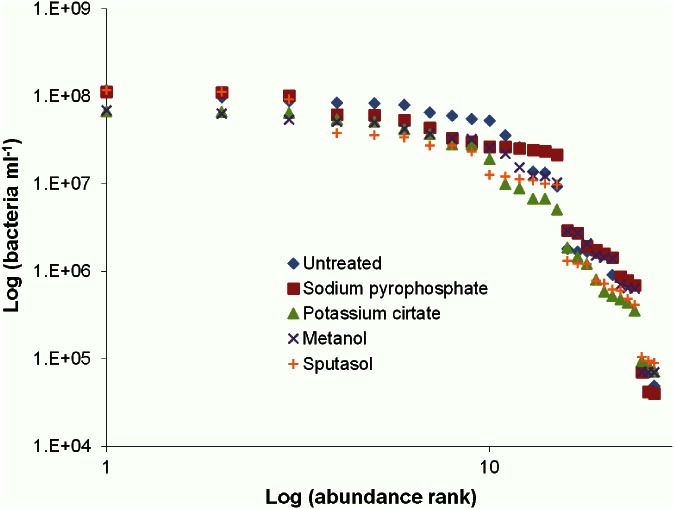
Rank abundance for each patient’s bacterial abundance for each sample treatment method, with triplicates shown. Three clear groups of patients with high, medium and low bacterial abundances are apparent. Differences in treatments used on the samples can be seen not to influence steps of bacterial abundance for patients.

**Fig 3:**
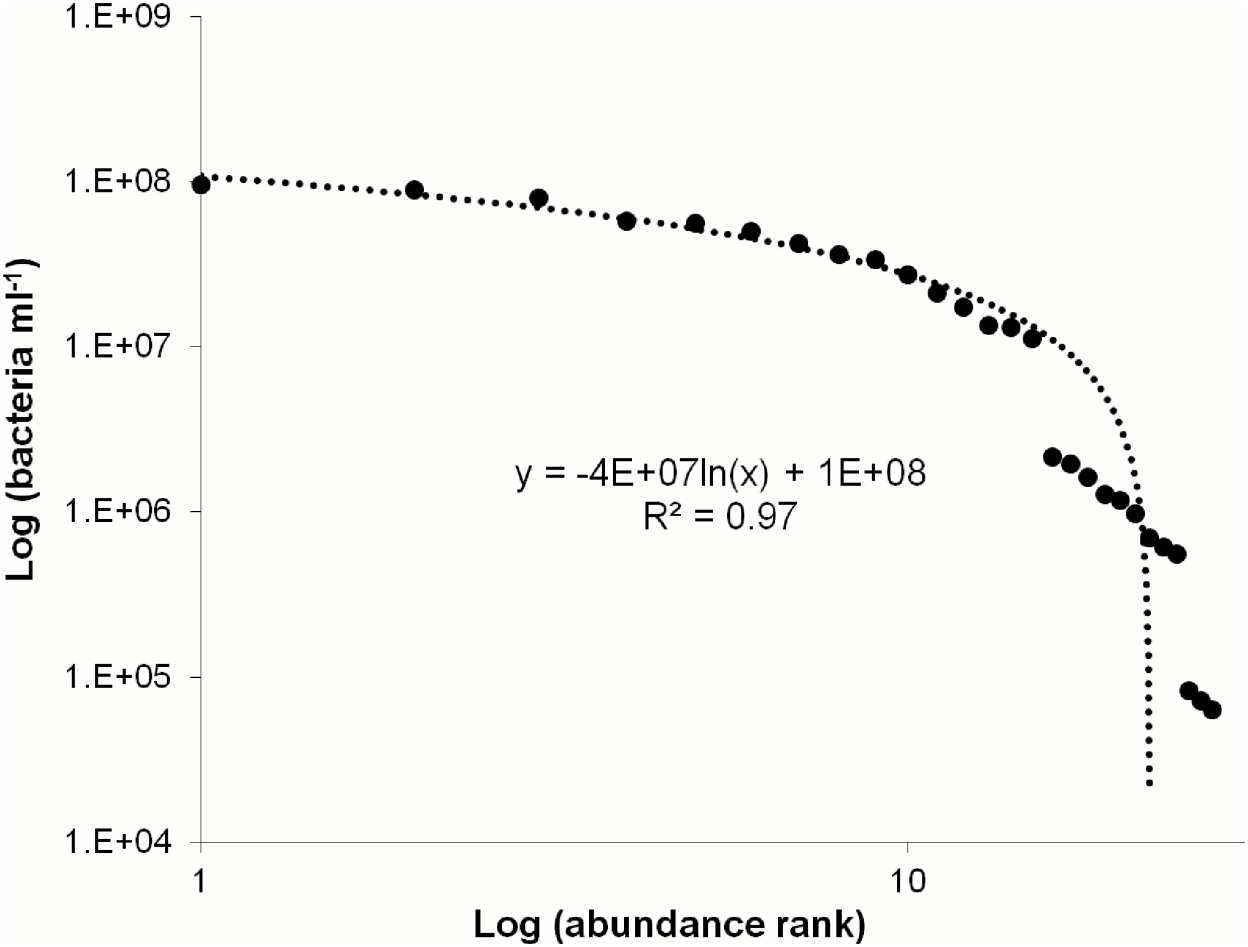
Average bacterial rank abundance using all treatment triplicates. Data points follow a logarithmic trend achieved by steps of power laws for each observed group. High medium and low bacterial concentration groups fit the power law equations y = 2E+08x^-0.84^ (R^2^ = 0.84), y = 5E+10x^-3.57^ (R^2^ = 0.98) and y = 6E+09x^-3.49^ (R^2^ = 1) respectively.

Rank abundance was used to identify possible groupings among the patient’s bacterial abundance. Breaks in the plot suggest 3 groupings of patients with bacterial abundances classified as high, greater than 10^7^ cells ml^-1^, medium, between 10^5^ to 10^6^ cells ml^-1^, and low, less than 10^5^ cells ml^-1^ (Fig 2). The high bacterial group consisted of triplicates from 5 patients. The medium bacterial group contained triplicates from 3 patients and the low bacterial abundance group consisted of 1 patient. There was approximately one order of magnitude difference between each of the 3 groups (Fig 2). The overall average bacterial rank abundance using all treatment triplicates fit a logarithmic trend; however this was achieved by a series of step down power laws for each bacterial group (Fig 3).

### VLP Rank Abundance

**Fig 4:**
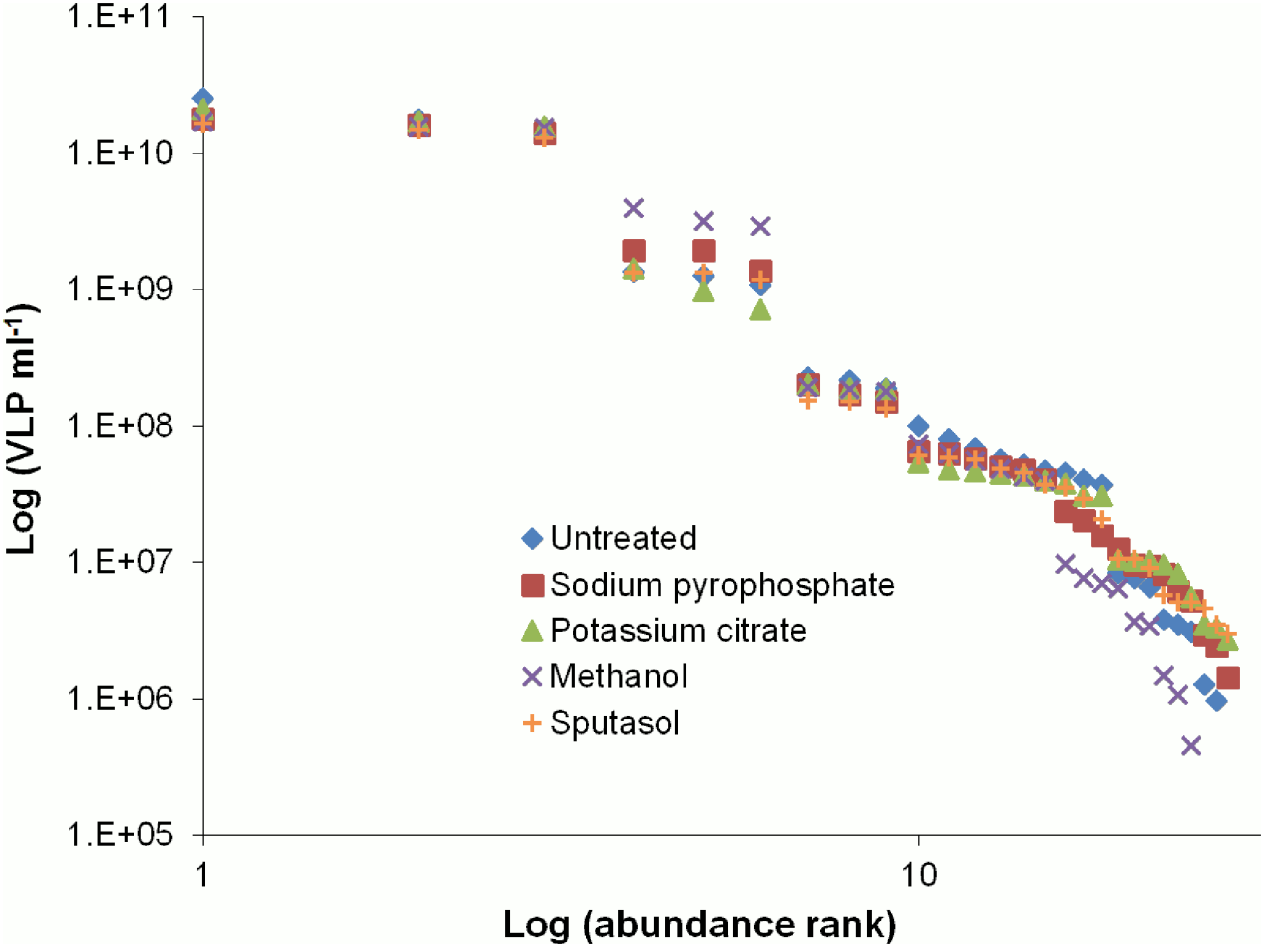
Rank abundance for each patient’s VLP abundance for each sample preparation method, with triplicates shown. Highest abundances are rare while lower abundances are common. Differences in treatments used on the patient samples can be seen not to influence the high range of VLP origination levels.

**Fig 5:**
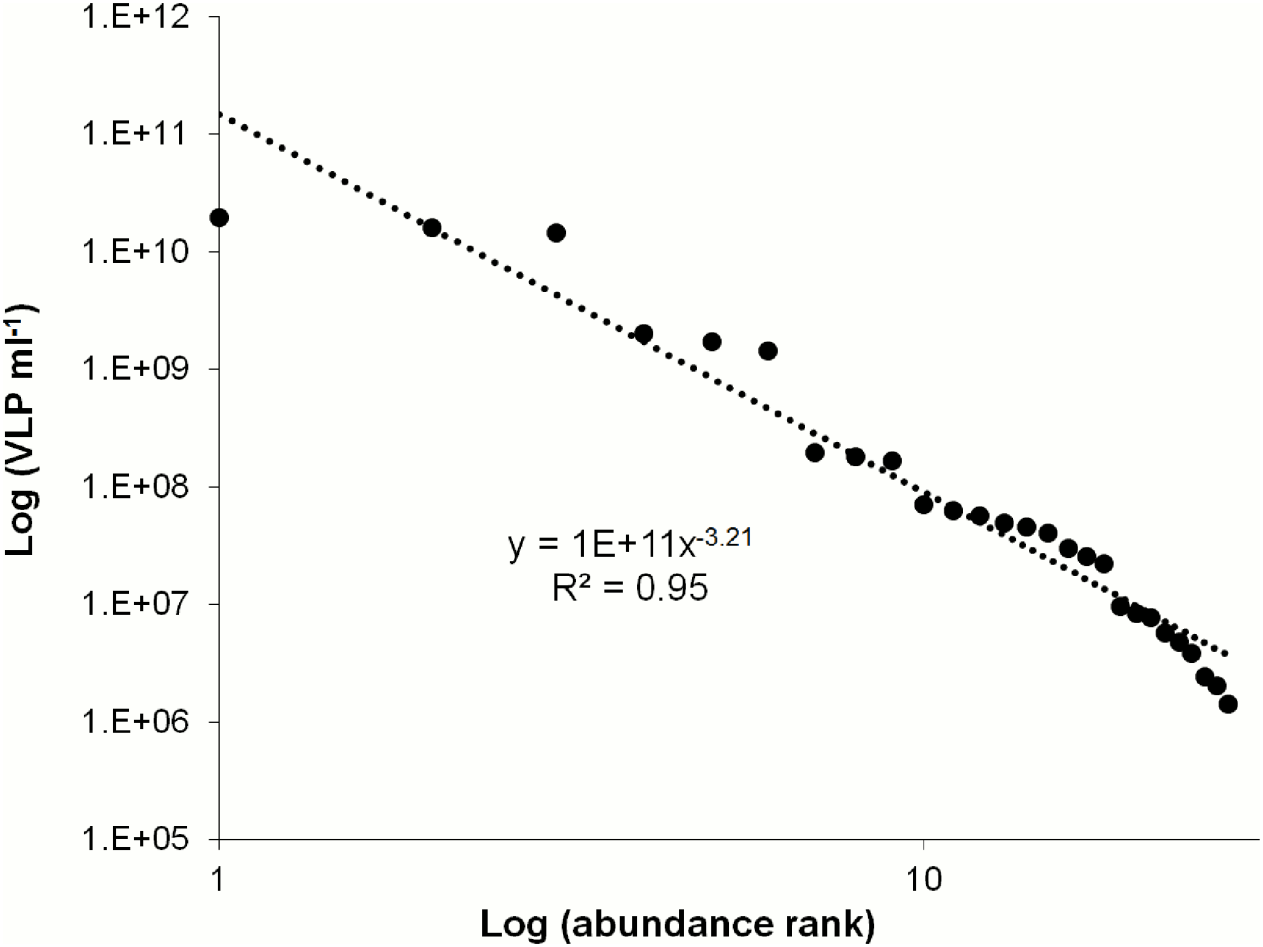
Average VLP rank abundance using all treatment triplicates. Data points follow a power law.

A rank abundance plot for VLP abundance was generated using the method triplicates of untreated, sodium pyrophosphate, potassium citrate, Sputasol and methanol samples (Fig 4). VLP abundances show an even distribution across 5 orders of magnitude. Three patients had values above 10^8^ VLP ml^-1^. These are classified as high VLP, with the proviso that they are separated from each other by an order of magnitude (Fig 4). Three patients make up the medium concentration group between 10^7^ and 10^8^ VLP ml^-1^. However, in this group the loss of VLPs in the methanol and sodium pyrophosphate treatments makes the group appear fused with the lowest group. The lowest group was classified as concentrations below 10^7^ VLP ml^-1^. In this group the methanol treatment showed considerable loss of VLPs and in one patient a complete absence of VLPs (Fig 4). The overall average VLP rank abundance using all treatment triplicates fit a steep power law (Fig 5).

### Lack of Correlation with Patient Symptoms

Prior to sinus surgery, each patient completed a questionnaire regarding basic clinical information and provided a severity score from 0, being no problem, to 5, being a problem as bad as it can be, for CRS symptoms for the past two weeks. Based on the rank abundance plots (Figs 2 and 3), possible trends in the patient groups were investigated. There were no trends observed within the rank abundance patient groups for bacteria or VLPs.

## Discussion

This is the first study to use flow cytometry to enumerate bacteria and VLPs within the maxillary sinus of CRS patients. We present a number of snapshot enumerations, using flow cytometry, of the microbial composition of sinuses of CRS patients. We tested a number of sample preparation techniques for bacterial and VLP enumeration that are used for environmental samples, in particular, techniques used for disruption of coral mucus, for microscopy [24, 27]. Our results showed that the sinus, at least in patients requiring sinus surgery, is an active microbiological environment. We speculate that most VLPs detected are likely to be bacteriophages as they are the most commonly found in association with their hosts, bacteria, which we find to be present in abundance in sinuses. The proposal that phages can be used to treat bacterial infections of the sinus [17] can now be viewed in the light of this data showing that phages appear to be present in sinuses in large numbers (Table 1).

Although the primary focus of this study was to enumerate the bacteria and VLPs in the sinus fluid, there was concern surrounding the presence of small fragments of mucus or biofilms within the samples prepared for flow cytometry. The mucus may have caused the bacteria and VLPs to clump together resulting in an overestimation on particle size, shape and DNA content. This is a similar concern in regards to analyzing bacteria and VLPs in coral mucus using microscopy [24, 27]. As bacterial and VLP flow cytometry on human samples is a new approach, various flow cytometry methods were investigated.

Common chemicals used in environmental sample preparation include potassium citrate, sodium pyrophosphate and methanol. Sodium pyrophosphate and potassium citrate are commonly used in environmental microbiology for desorbing viral particles from soil and marine sediment [28, 29]. Potassium citrate increases the electrostatic repulsion between viruses and bacteria, and the mucus to which they are attached to by raising pH [24]. Sodium pyrophosphate weakens the hydrophilic links in mucus allowing for viruses and bacteria to be separated [30]. Dithiothreitol (DTT) has been used on sinus samples to improve the yield of fungal cultures and in studies involving quantification of inflammatory cells in nasal secretions [31–33]. Sputasol contains DTT and has been used to liquefy mucus in nasal lavage [34]. It does this by breaking disulfide bonds within mucin, causing liquefaction, releasing trapped viruses and bacteria [35, 36]. Methanol has the ability to break up exopolymeric substances in mucus which entrap bacteria and viruses [37].

Our results show that the untreated and sodium pyrophosphate treatment methods yielded significantly higher bacterial abundances than all other methods tested (p < 0.05). For VLP enumeration, no treatment (as in the untreated samples) was the optimal method. Although there was no significant difference between untreated and sodium pyrophosphate treated samples (p < 0.05), sodium pyrophosphate did not yield significantly higher VLP abundances than methanol and potassium citrate (p < 0.05). This result contrasts to previous microscopy studies which found potassium citrate better for viral enumeration in coral mucus [24]. Unlike sample preparation for coral mucus for microscopy, flow cytometry includes an incubation period after addition of SYBR Green. During this step viral capsids may partially and temporarily denature, facilitating a greater uptake of SYBR Green [20]. This may result in brighter VLP fluorescence resulting in higher counts for all samples. It is also possible that the extra processing steps involved with each treatment resulted in the loss of bacteria and VLPs. All samples were diluted for flow cytometry in TE buffer which contained 1 mM of EDTA. EDTA has been used to extract bacteria and viruses from photosynthetic microbial mats as it destroys cation links within exopolymeric substances, releasing bound bacteria and viruses [38]. It also permeabilises outer membranes, facilitating greater uptake of SYBR Green [39]. Additional treatment to each sample could have an adverse effect, causing damage to viral capsid proteins or to bacterial cell walls. Our data suggests that the least possible number of processing steps and addition of chemicals is the optimal method for analyzing sinus flush samples.

Previous studies have shown that the human sinus is colonized with an array of microbes [1, 2, 6–12, 40]. Flow cytometry enables categorization and enumeration of these microbes based on size and DNA content [21, 26]. Some patient’s cytograms revealed numerous bacterial sub-populations (Fig 1). These populations displayed increased green fluorescence, an indication of DNA content, and size. These different sub-populations may be reflecting different bacterial replication stages or are indicative of different bacterial species with different sized genomes. In the Sputasol treated patient samples, an unusual population was observed between the bacterial and VLP regions. This population appears to be an artifact of the Sputasol as it was also observed in the control samples (S1 Fig). Thus, there may be a component within Sputasol which autofluorescences or binds to SYBR Green.

Large variations were observed between patients bacterial and VLP concentrations, which is not uncommon with human microbial flora studies [41–45]. These findings suggest that the maxillary sinus is either extremely dynamic or highly individualised. These differences in the patient microbial concentrations could indicate the need for personalised dosages when treating CRS with antibiotics or with phage. The rank abundance plot for patient bacterial abundance revealed 3 groupings of high, medium and low abundance (Fig 2). As the treatments used on the samples were not seen to account for the differences in bacterial and VLP levels (Figs 2 and 4), an average of all treatment values was used to clearly demonstrate the obvious trends in bacterial concentration groups (Fig 3) and high episodic nature of the VLPs (Fig 5).

For the bacterial rank abundance, the three orders of magnitude range among the 9 patients may reflect temporal variation or that the bacterial abundances are defined by processes or a variable that was not measured. The skew of the distribution towards the lower concentrations in the VLP rank abundance is consistent with the highly episodic nature of viral infections, particularly in bacteriophage where large burst sizes can quickly reduce the concentrations of particular bacterial species, leaving high bacteriophage concentrations at least temporarily [46]. From this, one might posit that time series sampling would show an eventual decline in VLP concentration. While bacteriophage dynamics is one likely explanation for the observed distribution, at this point we cannot discount that some patients have chronically high VLP concentrations. To investigate this and its clinical significance would require flow cytometric and nucleic acid sequence analysis of time series samples from the identified patients. Due to the invasive nature of sampling used in this study, healthy controls and a time series using the same sampling method may not be a feasible option. Therefore there is the need to develop a proxy for an alternative less invasive sampling strategy, such as nasal swabbing. While this is beyond the scope of this paper, it is a potentially valuable future direction.

Knowledge of the abundance of microorganisms in CRS will further our understanding of the disease as the presence of certain bacterial species does not always imply infection. The aim of his research was to produce a snapshot enumeration of the sinus microbes of 9 patients known to suffer from CRS, and to determine if they had similar abundances of bacteria and VLPs. Within the group of 9 patients sampled, there was 3 orders of magnitude difference in abundance for bacterial populations and almost 4 orders of magnitude difference for VLPs. This suggests that not all CRS patients are infected at the same level of bacteria and VLPs. Knowledge of the differences in bacterial abundances may facilitate the development of personalised treatment options.

This work indicates the potential for future studies in other microbial disease related health conditions. We propose that flow cytometry has potential as a tool to monitor microbial dynamics in patients and in future may assist in determining appropriate dosages required when treating microbial related health conditions.

## Supporting Information

S1 Fig Representative cytogram showing the Sputasol artefact population observed between the VLP and bacterial populations. (TIF)

S1 Table Patients total bacterial abundances for each optimisation method. Replicates (Rep) for each method are shown.
(PDF)

S2 Table Patients total VLP abundances for each optimisation method. Replicates (Rep) for each method are shown.
(PDF)
