# Supplementary figures and images for: Enumerating Virus-Like Particles and Bacterial Populations in the Sinuses of Chronic Rhinosinusitis Patients Using Flow Cytometry
Source: PLoS One. 2016 May 12;11(5):e0155003. doi: 10.1371/journal.pone.0155003 (PMC4865123; doi:10.1371/journal.pone.0155003)

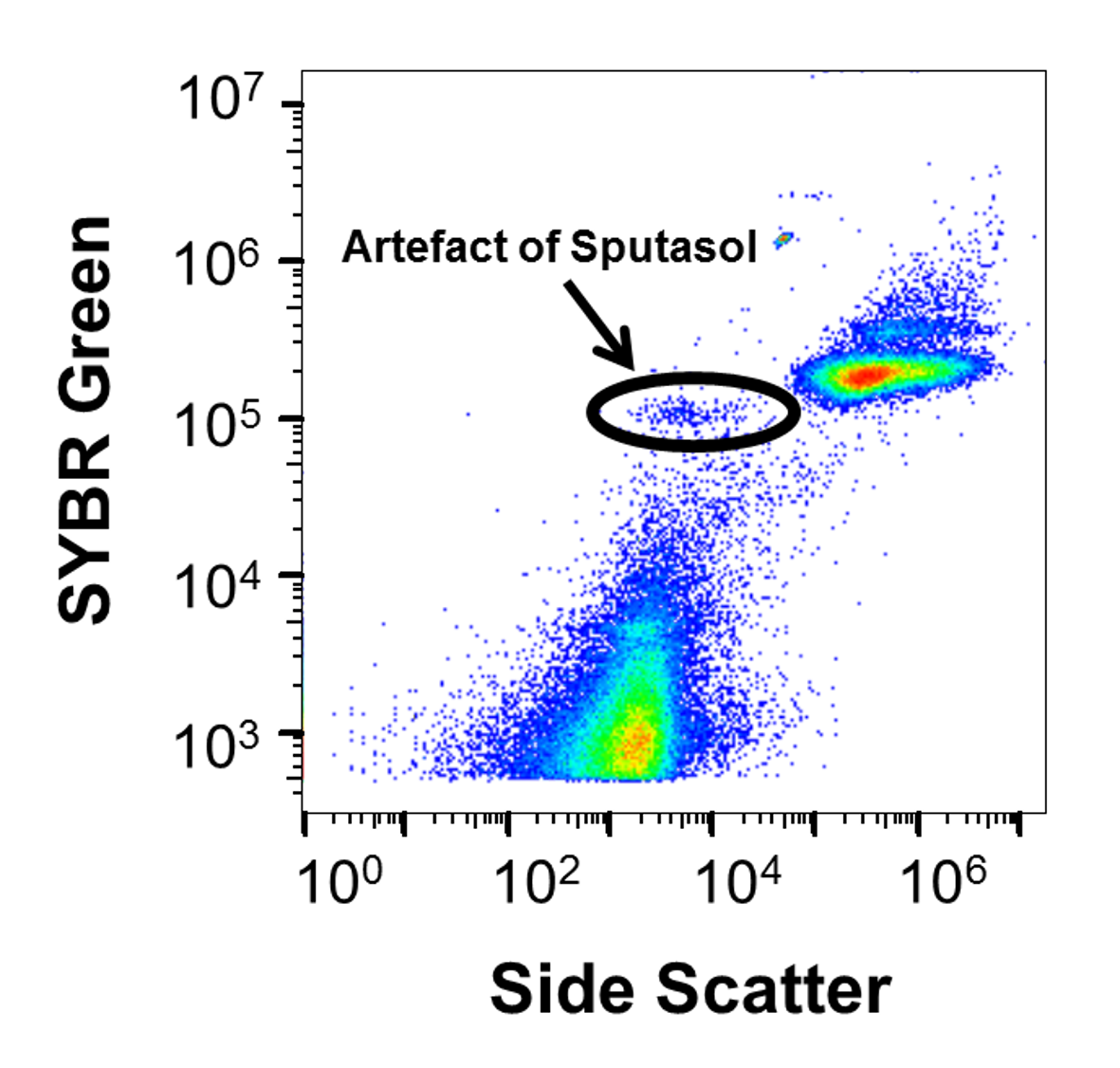

Supplement: S1 Fig — (TIF) [file pone.0155003.s001.tif]
