# Supplementary material for: Enumerating Virus-Like Particles and Bacterial Populations in the Sinuses of Chronic Rhinosinusitis Patients Using Flow Cytometry
Source: PLoS One. 2016 May 12;11(5):e0155003. doi: 10.1371/journal.pone.0155003 (PMC4865123; doi:10.1371/journal.pone.0155003)
Supplement: S1 Table — Replicates (Rep) for each method are shown. (PDF) [file pone.0155003.s002.pdf]

S1 Table. Patients total bacterial abundances for each optimisation method. Replicates (Rep) for each method are shown.

	**Untreated**			**Sodium pyrophosphate**			**Potassium citrate**			**Methanol**			**Sputasol**		
	Rep 1	Rep 2	Rep 3	Rep 1	Rep 2	Rep 3	Rep 1	Rep 2	Rep 3	Rep 1	Rep 2	Rep 3	Rep 1	Rep 2	Rep 3
**Patient 1**	1.82E+06	1.60E+06	1.62E+06	6.94E+05	8.73E+05	7.93E+05	3.54E+05	4.75E+05	4.39E+05	2.10E+06	2.92E+06	2.76E+06	1.32E+06	1.22E+06	1.24E+06
**Patient 2**	8.63E+05	7.33E+05	6.99E+05	1.45E+06	1.94E+06	1.75E+06	5.20E+05	5.83E+05	7.98E+05	1.44E+06	1.41E+06	1.52E+06	6.18E+05	4.89E+05	4.17E+05
**Patient 3**	8.52E+07	1.17E+08	9.83E+07	1.02E+08	1.11E+08	1.12E+08	4.21E+07	5.18E+07	5.42E+07	4.29E+07	3.77E+07	5.15E+07	3.80E+07	3.61E+07	3.38E+07
**Patient 4**	6.91E+04	4.92E+04	7.96E+04	6.98E+04	4.00E+04	4.23E+04	9.37E+04	8.53E+04	7.17E+04	6.99E+04	6.99E+04	7.10E+04	1.05E+05	9.43E+04	9.00E+04
**Patient 5**	1.69E+06	1.67E+06	9.17E+05	1.59E+06	2.73E+06	2.96E+06	1.22E+06	1.86E+06	1.45E+06	6.32E+05	7.08E+05	6.42E+05	7.22E+05	6.03E+05	7.75E+05
**Patient 6**	8.84E+07	6.54E+07	5.56E+07	2.36E+07	2.68E+07	4.40E+07	2.84E+07	2.82E+07	3.72E+07	1.55E+07	3.30E+07	5.47E+07	1.13E+07	1.21E+07	1.01E+07
**Patient 7**	1.38E+07	9.32E+06	1.33E+07	3.03E+07	3.35E+07	2.17E+07	1.91E+07	6.71E+06	8.86E+06	5.06E+07	6.38E+07	6.96E+07	1.10E+07	1.26E+07	9.79E+06
**Patient 8**	6.04E+07	8.00E+07	8.42E+07	6.09E+07	5.39E+07	6.24E+07	6.75E+07	6.65E+07	6.52E+07	3.25E+07	2.23E+07	2.61E+07	1.19E+08	1.13E+08	9.20E+07
**Patient 9**	3.59E+07	2.65E+07	5.31E+07	2.56E+07	2.44E+07	2.68E+07	9.96E+06	5.07E+06	6.70E+06	1.04E+07	1.24E+07	1.25E+07	2.76E+07	2.76E+07	2.35E+07
