# Supplementary material for: Enumerating Virus-Like Particles and Bacterial Populations in the Sinuses of Chronic Rhinosinusitis Patients Using Flow Cytometry
Source: PLoS One. 2016 May 12;11(5):e0155003. doi: 10.1371/journal.pone.0155003 (PMC4865123; doi:10.1371/journal.pone.0155003)
Supplement: S2 Table — Replicates (Rep) for each method are shown. (PDF) [file pone.0155003.s003.pdf]

S2 Table. Patients total VLP abundances for each optimisation method. Replicates (Rep) for each method are shown.

	Untreated			Sodium pyrophosphate			Potassium citrate			Methanol			Sputasol		
	Rep 1	Rep 2	Rep 3	Rep 1	Rep 2	Rep 3	Rep 1	Rep 2	Rep 3	Rep 1	Rep 2	Rep 3	Rep 1	Rep 2	Rep 3
**Patient 1**	1.00E+08	8.12E+07	6.86E+07	2.06E+07	2.39E+07	1.58E+07	4.51E+07	3.08E+07	4.68E+07	4.29E+07	6.35E+07	5.63E+07	2.92E+07	4.56E+07	3.74E+07
**Patient 2**	6.66E+06	7.76E+06	8.42E+06	8.23E+06	6.15E+06	9.61E+06	1.03E+07	1.04E+07	1.04E+07	3.70E+06	3.46E+06	6.48E+06	5.08E+06	2.99E+06	5.15E+06
**Patient 3**	3.75E+07	4.77E+07	4.09E+07	5.78E+07	6.60E+07	6.35E+07	3.09E+07	4.32E+07	3.99E+07	1.07E+06	4.60E+05	9.75E+06	6.13E+07	5.76E+07	5.95E+07
**Patient 4**	3.49E+06	3.85E+06	3.14E+06	2.46E+06	1.45E+06	2.94E+06	3.51E+06	3.36E+06	2.71E+06	0	0	0	5.80E+06	4.63E+06	3.52E+06
**Patient 5**	0	9.79E+05	1.27E+06	5.27E+06	9.45E+06	1.28E+07	5.60E+06	9.70E+06	8.24E+06	7.04E+06	1.48E+06	7.72E+06	9.16E+06	1.07E+07	1.06E+07
**Patient 6**	2.55E+10	1.78E+10	1.55E+10	1.43E+10	1.80E+10	1.63E+10	1.72E+10	1.57E+10	2.12E+10	1.57E+10	1.71E+10	1.55E+10	1.50E+10	1.30E+10	1.66E+10
**Patient 7**	1.26E+09	1.09E+09	1.36E+09	1.38E+09	1.95E+09	1.94E+09	7.27E+08	9.87E+08	1.42E+09	3.20E+09	4.03E+09	2.96E+09	1.18E+09	1.33E+09	1.34E+09
**Patient 8**	1.88E+08	2.28E+08	2.16E+08	1.52E+08	1.72E+08	2.02E+08	1.87E+08	2.05E+08	1.89E+08	1.87E+08	1.79E+08	1.93E+08	1.36E+08	1.51E+08	1.55E+08
**Patient 9**	5.22E+07	4.61E+07	5.67E+07	5.04E+07	4.86E+07	4.06E+07	4.86E+07	3.77E+07	5.41E+07	7.40E+07	4.92E+07	3.98E+07	3.55E+07	4.94E+07	2.09E+07
